# MicroRNA in Brain pathology: Neurodegeneration the Other Side of the Brain Cancer

**DOI:** 10.3390/ncrna5010020

**Published:** 2019-02-23

**Authors:** Jakub Godlewski, Jacek Lenart, Elzbieta Salinska

**Affiliations:** 1 Affiliation Department of Neurosurgery, Brigham and Women’s Hospital, Harvard Medical School, Boston, MA 02115, USA; jgodlewski@bwh.harvard.edu; 2 Affiliation Department of Neurochemistry, Mossakowski Medical Research Centre, Polish Academy of Sciences, 02-106 Warsaw, Poland

**Keywords:** microRNA, neurodegeneration, brain tumors, glioblastoma, brain ischemia, Alzheimer’s disease, Parkinson’s disease, Huntington’s disease

## Abstract

The mammalian brain is made up of billions of neurons and supporting cells (glial cells), intricately connected. Molecular perturbations often lead to neurodegeneration by progressive loss of structure and malfunction of neurons, including their death. On the other side, a combination of genetic and cellular factors in glial cells, and less frequently in neurons, drive oncogenic transformation. In both situations, microenvironmental niches influence the progression of diseases and therapeutic responses. Dynamic changes that occur in cellular transcriptomes during the progression of developmental lineages and pathogenesis are controlled through a variety of regulatory networks. These include epigenetic modifications, signaling pathways, and transcriptional and post-transcriptional mechanisms. One prominent component of the latter is small non-coding RNAs, including microRNAs, that control the vast majority of these networks including genes regulating neural stemness, differentiation, apoptosis, projection fates, migration and many others. These cellular processes are also profoundly dependent on the microenvironment, stemness niche, hypoxic microenvironment, and interactions with associated cells including endothelial and immune cells. Significantly, the brain of all other mammalian organs expresses the highest number of microRNAs, with an additional gain in expression in the early stage of neurodegeneration and loss in expression in oncogenesis. However, a mechanistic explanation of the concept of an apparent inverse correlation between the odds of cancer and neurodegenerative diseases is only weakly developed. In this review, we thus will discuss widespread de-regulation of microRNAome observed in these two major groups of brain pathologies. The deciphering of these intricacies is of importance, as therapeutic restoration of pre-pathological microRNA landscape in neurodegeneration must not lead to oncogenesis and vice versa. We thus focus on microRNAs engaged in cellular processes that are inversely regulated in these diseases. We also aim to define the difference in microRNA networks between pro-survival and pro-apoptotic signaling in the brain.

## 1. Background

The last twenty years dramatically changed the simplified view that protein coding genes are hugged by an ocean of non-transcribed sequences. Now, the view is that protein coding genes may act largely thanks to a number of non-coding transcripts that regulate their expression, translation or activity [1]. Most of these non-coding RNAs have still unknown function. However, a small fraction of the non-coding genome gives rise to small regulatory RNAs, such as microRNAs [2], So far more than 2500 have been identified in the human genome (miRBase (www.mirbase.org)) [3] and hundreds have already been described to be functional in the brain development and pathology.

MicroRNAs are transcribed as long, single-stranded primary transcripts which form a hairpin loop structure—a signal for RNA nuclease cleavage resulting in short hairpin precursor-microRNA. Precursor-microRNAs undergo cleavage into 17–22 nucleotide-long mature microRNAs. Many microRNAs are highly conserved in other vertebrate animals. However, although they are conserved in sequence they, as other non-coding RNA, are not conserved in transcription patterns. As they regulate levels of their target messenger RNA (mRNA) through a combination of rapidly occurring mRNA destabilization and translational repression, they are potent regulators of protein expression but also other non-coding RNAs. Moreover, a single microRNA is capable of regulating hundreds of mRNA species, making them important regulators of cellular homeostasis [4]. MicroRNAs initiate the formation of inhibitory complexes by binding to partially complementary target regions within the 3′ untranslated region (3′ UTR) of specific mRNAs [5] but other mechanisms, including targeting the 5′ untranslated region (5′ UTR) or coding sequence (CDS) of the targeted mRNA, have also been reported [6,7,8,9,10,11].

Scientists have long noted an inverse correlation between the likelihood of cancer and neurodegenerative diseases. However, only scant data puts meat on the bones of that idea. Even less blended data, represent a trade-off between cancer and neurodegeneration in the context of microRNA. Inspired by a research group led by Kaleta et al. [12] who reported that the transcriptional changes seen in aging people and other vertebrates are analogous to those that occur in neurodegeneration but dissimilar from changes in cancer, we asked whether that is also true in case of microRNA. It will be essential to ensure that targeting neurodegeneration drivers or executor genes including non-coding ones do not raise cancer risk, especially in the context of a recently rapidly developing strategy of immunotherapy (as anti-inflammatory statins, for example, have been found to boost the odds of cancer) [13,14,15,16,17].

## 2. Neurodegeneration

The mammalian brain is a complex structure, made up of billions of neurons and glial cells, intricately connected [17]. Perturbations on synaptic connections can lead to neuronal degeneration by progressive loss of structure and malfunction of neurons, including death. The neurodegeneration is irreversible, and so far, there are no effective methods to prevent initiated processes.

Neurodegeneration is a large group of various disorders driven by a complex genetic and environmental determinant. Alzheimer’s disease (AD), Parkinson’s disease (PD) or Huntington’s disease (HD), the most often occurring neurodegenerative diseases have been the subject of investigations for many years, and many of the aspects have been disclosed [18]. Valuable information concerning the molecular mechanisms implicated in neurodegenerative diseases is related to the discovery of familial forms of these diseases, which are inherited because of specific mutations. Accumulation of aberrant or misfolded proteins, protofibril accumulation, and dysfunction of the ubiquitin-proteasome system are the commonly known landmarks of these diseases. The development of excitotoxicity, oxidative and nitrosative stress, mitochondrial injury, disturbances in synaptic transmission and inefficient axonal and dendritic transport has been reported in many slowly progressing neurodegenerative disorders [19].

Neurodegeneration can also develop after ischemic and hypoxic conditions like stroke or birth asphyxia and after accidents resulting in traumatic brain injury (TBI). Brain ischemia is a restriction in blood supply to the brain, causing not only a shortage of oxygen but also the main energy supplier—glucose. Insufficient blood supply may result from cardiac arrest (global ischemia) or can be an effect of stroke (local ischemia). In these cases, the neurodegeneration occurs for a short time in the ischemic core zone but may also slowly develop in the ischemic penumbra [20].

In the hypoxic/ischemic insults, the most critical factors are over-activation of ionotropic glutamate receptors, especially N-methyl-D-aspartate (NMDA) receptor resulted from an increased release and extracellular retention of glutamate leads to the accumulation of toxic products (e.g., reactive oxygen species (ROS) resulting from insufficient oxygen and glucose supply) [21,22]. The shift of the cellular balance between the production and scavenging of ROS in favor of oxidants initiates oxidative stress. Cells are equipped in antioxidant enzymes such as superoxide dismutase (SOD), catalase (CAT) and glutathione peroxidase (GPx) supported by glutathione that eliminates ROS to combat oxidative stress and to neutralize reactive oxygen species; however, in ischemic conditions, these weapons usually appear insufficient.

## 3. Brain Cancer

Glioblastoma multiforme (GBM) has a particularly poor prognosis of only 18 months and a mean survival rate of only 3.3% at two years and 1.2% at three years [23,24,25]. This primary brain cancer is the most aggressive and lethal while being notoriously insensitive to radiation and chemotherapy. One of the reasons why GBM is so difficult to treat is the presence of extensive cellular and genetic heterogeneity, even at the single-cell level. [26,27,28]. GBM tumors are also highly invasive, infiltrating surrounding brain tissue, which makes it impossible to fully resect. Although GBM cells have distinct phenotypes, genotypes, and epigenetic landscapes, analyses on molecular diversity have focused mostly on protein-coding transcripts [28]. The global engagement and contribution of non-coding RNAs are still not sufficiently studied [29]. Recent studies showing that long-non-coding RNA [30,31] but not microRNA [32] can be used for subtype classification in GBM, underline the complexity of this cancer.

## 4. MicroRNA in the Brain

Multiple lines of evidence reveal several distinct mechanisms by which microRNA regulatory pathways contribute to human brain development and disease. The highly dynamic changes in microRNA expression support the emerging view that many microRNAs are expressed in cell-type-specific patterns [32,33]. Thus cell-type context may play an essential role in microRNA/mRNA targeting.

To gain insight into the function of microRNA in the pathophysiology of the brain, microRNAome profiling in neurodegeneration and oncogenesis was analyzed. This indicated apparent cell-type-specific function, as many microRNAs, including both cell/tissue-specific as well as those commonly expressed in multiple cell types, regulate the expression of cell type-specific genes. The brain, of all other mammalian organs, expresses the highest number of microRNAs [34] and therefore they have been considered as essential modulators of many brain functions in both physiological and pathological conditions. The modulatory potential of microRNAs is additionally increased by their multitargeting abilities, as one microRNA can control the expression of multiple genes.

A changed expression pattern of microRNAs has been observed in neurodegenerative disorders, ischemic brain areas and in brain tumors. The developing knowledge on the role of microRNAs in pathology and understanding of their impact on neurogenesis and neuroprotection make them a potential target for new therapeutic applications [35]. However, our understanding of the underlying cellular mechanisms of most neurological disorders is limited, as deregulated neuronal cells, likely to be the most informative, are already lost. Conversely, undifferentiated cancer stem cells are potent enough to self-renew from a single cell and therefore are an excellent source of information on disease etiology.

Therefore, pathobiology of microRNA in cancer research may allow the identification of targets for cancer and neurodegeneration leading to improved treatments for both disorders [36]. The involvement of microRNAs in the pathophysiology of neurodegeneration and brain tumors is a field of study of broad and current interest and the number of reported findings is rapidly rising and include different aspects of mentioned disorders.

## 5. Brain Pathology and microRNAs

Cancer and neurodegenerative diseases are influenced by common signaling pathways regulating the balance of cell survival and death [37]. Thus, the molecular machinery involved in maintaining neural function in neurodegenerative diseases may be shared with oncogenic pathways. Several microRNAs differentially expressed in neurodegenerative diseases are considered to be potential tumor suppressor microRNAs (Table 1). Indeed, cancer and neurodegenerative disorders may be influenced by common microRNA pathways that regulate differentiation, proliferation, and death of cells.

### 5.1. miR-9 and REST Network

Non-coding RNA transcripts are often deregulated by aberrant DNA methylation at CpG island promoters. Such a mechanism is also responsible for *miR-9* (*miR-9* and miR-9*) downregulation in various human cancers including brain cancers, suggesting that miR-9 is a potential tumor suppressor microRNA [38]. Indeed, *miR-9* restoration in mutant EGFR driven glioma cells [39] downregulates its target FOXP1 and decrease tumorigenicity. *MiR-9* is part of a feedback loop that allows tight control of the expression levels of target genes that coordinate the proliferation and migration of GBM cells [40]. In contrast to increasing colony numbers of glioblastoma stem cells via CAMTA1, *miR-9* has been shown to inhibit proliferation of non-stem cell lines. Mechanistically *miR-9* inhibits the proliferation and promotes the migration of glioma cells by directly targeting cyclic AMP response element-binding protein (CREB) and neurofibromin 1 (NF1), respectively [40,41,42,43]. Reduction in proliferation and tumor growth by *miR-9* at the molecular level was shown to be associated with its targeting of stathmin (STMN1). On the cellular level *miR-9*-STMN1 targeting regulated microtubule formation during cell-cycle progression. [44]. Taking into account the complexity of the heterogeneous population of cancer cells in GBM, the majority of data still needs validation by using multiple patient-derived cells with diverse transcriptome and phenotype characteristics. The *miR-9* expression is also decreased early in HD, targeting two components of the REST complex (*miR-9* targets REST and miR-9* targets CoREST) [45]. *MiR-9* transiently increases after brain injury and is required for axon regeneration [46]. Ectopic expression of *miR-9/9^∗^* (but also *miR-124*) in adult human fibroblasts has been found to evoke extensive reconfigurations of the chromatin and direct the fate conversion to neurons. The *miR-9*-dependent repression of the EZH2-REST axis opens chromatin regions harboring REST binding sites and in consequence shapes the neuronal program [47]. *MiR-9* regulates adult neurogenesis thus serving as a negative regulator providing a balance between neural stem cells (NSC) proliferation and differentiation. However, its upregulation and in consequence pro-apoptotic function was also described in PD and AD pathology. Targeting SIRT1 and BACE1 by *miR-9* can affect not only on cell survival but also oxidative stress response [48,49]. The conversion of somatic cells into neurons holds great promise for regenerative medicine [50]; it is also premise in targeting cancer stem cells into the differentiative stage, and *miR-9* can be one of the gatekeepers that enable deterministic reprogramming of undifferentiated cells into functional neurons [50]. The mechanisms by which miR-9/9* drive oncogenesis and neurodegeneration underline the cellular context in which these microRNAs operate [40].

### 5.2. miR-29 Family-Methyltransferases and Cell Death

The *miR-29* family (*miR-29a, miR-29b*, and *miR-29c*) was shown to target the de novo DNA methyltransferases DNMT3A and DNMT3B. Its expression is suppressed in brain cancer cells, both differentiated and stem-like.

As a consequence of the reduced expression of the *miR-29*, DNMT3A and DNMT3B are overexpressed. That in turn results in aberrant DNA methylation in glioblastoma and other cancers [37,51,52,53]. *MiR-29* inhibits invasion and proliferation of glioblastomas due to targeting podoplanin membrane sialoglycoprotein encoded by PDPN gene were also demonstrated [54]. Preventing de novo methylation of DNA is an important cellular anti-tumorigenic strategy. However, the described opposite result in global DNA methylation level due to overexpression of *miR-29* in different cancer cell types suggested that *miR-29* suppresses tumorigenesis by protecting against changes in the existing DNA methylation status [55]. Thus, the firmly established tumor suppressive function of *miR-29* needs to be taken into account as the cell-specific transcriptome to understand the contrast between its anti-tumorigenic function and targeting of potent tumor suppressor PTEN [56].

There are endless discussions between cancer researchers and neuroscientists on how PTEN mutated in cancer and deregulated in neurodegeneration [57] drive opposite cellular fates. Although patients with neurodegenerative illness are generally not more susceptible to cancer, PD patients do show an increased risk for brain tumors, suggesting that context matters, and additional alterations are required for full-blown malignant transformation. A down-regulative correlation of *miR-29a/b* with neurodegenerative disease conditions was shown in both AD and HD [58]. Downregulation of *miR-29* in AD patient samples shows an association with the upregulation of β-secretase 1 (BACE1) enzyme, which contributes to the formation of plaques by cleavage of the amyloid precursor protein (APP) [59]. Significantly, the down-regulation of *miR-29* expression was also shown in the cellular model of spinocerebellar ataxia 17 (SCA17) [59]. In primary neurons and the adult mouse brain, *miR-29* is highly expressed [60]; its expression is activated during cerebral and cortical maturation. The expression of *miR-29* in the sympathetic nervous system inhibits apoptosis and loss of *miR-29* results in neuronal cell death and ataxia implicating its function in brain pathology [58].

### 5.3. miR-34abc–Link to p53

One of the most broadly studied proteins in carcinogenesis, which has been recently shown to be also involved in neurodegeneration, is p53 [61,62]. *MiR-34a* was identified as a target of p53. It induces a cell cycle arrest, senescence, and apoptosis [63,64]. Aberrant CpG methylation of *miR-34* promoter results in its loss in GBM similar to other cancers types [65,66,67,68,69]. However, its re-introduction may have a diverse effect depending on cancer driver oncogene. Indeed, overexpression of *miR-34a* in a subset of glioblastoma cells highly expressing PDGFRA, but not EGFR, resulting in reduced cell growth [65].

Proteins controlling the cell cycle are de-regulated in neurodegeneration. This leads to the hypothesis that, in senescent neurons, aberrations in proteins engaged in cell cycle control and apoptosis affect neuronal plasticity [65]. Terminal differentiation of neurons must be synchronized with cell cycle suppression. However, neurons can also reenter the cell cycle and undergo DNA replication [70,71,72,73].

The analysis of microRNA expression showed the downregulated profile of *miR-34bc* associated with pathological brain tissue in different clinical stages of PD [37,74]. Although reduction of *miR-34bc* expression in differentiated dopaminergic neuronal cells results in a moderate reduction in cell viability, it was accompanied by altered mitochondrial function [75]. DJ1 and Parkin were also shown to be *miR-34bc* indirect targets [74].

Pathogenesis of AD is also linked with the disrupted cell cycle. *MiR-34a* prevents cell cycle reentry and suppresses the neuronal cell cycle by targeting cyclin D1 [76]. As a consequence of *miR-34a* expression in cortical neurons treated with neurotoxic Aβ42 and neurons from a transgenic mouse model for AD, the aberrant increase in cyclin D1 levels occurs leading to cell cycle reentry and apoptosis of neurons. Earlier studies have shown that *miR-34a* is a requisite for proper neuronal differentiation, partially by targeting SIRT1 and modulating p53 activity [77,78]. p53 is known to regulate the expression of microRNAs globally either by regulating transcription of microRNAs directly or by regulating microRNA processing, and maturation machinery p53 regulate the expression of *miR-34a* by acting on specific binding sites on the *miR-34a* promoter [70]. These findings suggest that p53 may play an important role in the initiation and progression of both AD and PD via various *miR-34* mediated mechanisms.

### 5.4. miR-124–Differentiation and Neurogenic Niche 

*MiR-124* is the most abundant neuronal microRNA. Biogenesis of *miR-124* has discernible spatiotemporal profiles in various cells and tissues and affects a broad spectrum of biological functions in the central nervous system including its pathologies (reviewed in References [77,79]). During central nervous system development, *miR-124* expression gradually increases and accumulates as neurons maturate [80,81,82]. Inhibition of *miR-124* activity in selectively mature neurons leads to increased levels of non-neuronal transcripts [79,83] while increasing *miR-124* activity in cancer cells showed a shift of expression profile toward that of neuronal lineage [77]. Re-introduction of *miR-124* to brain cancer cells is responsible for induced morphological changes as reduced self-renewal, tumorigenicity and inhibition of invasion [84,85] by targeting SCP1, PTPN12, SNAIL2, and ROCK1, [86,87]. Such phenotype is mostly dependent on cell type, driver oncogene, and stemness status, as other report noted *miR-124*-dependent glioblastoma differentiation by suppressing TWIST and SNAI2 [88]. Re-expression of *miR-124* in vivo increases cell death and survival of mice with intracranial xenograft tumors. *MiR-124* exerts this phenotype in part by direct regulation of TEAD1, MAPK14/p38α, and SERP1 that are involved in cell proliferation and survival under stress [89].

In neurodegenerative disorders *miR-124* was shown to be involved in AD, PD, and HD, as in all cases it is significantly downregulated, but in contrast to cancer cells, its low level alleviates cell death. The modulation of the subventricular zone (SVZ) neurogenic niche by *miR-124* enhanced brain repair in PD model. Delivery of this microRNA is associated with the downregulation of Sox9 and Jagged1 expression, two *miR-124* targets and stemness-related genes [90]. Inhibition of apoptosis in a model of PD was shown to reduce the loss of DA neurons by targeting pro-apoptotic BIM by *miR-124* [91]. Widespread depression of neural genes in the caudate and motor cortex, including *miR-124*, is characteristic for HD. During neuronal differentiation, *miR-124* plays the role of a repressor of the non-neuronal form of the splicing factor, PTB [92]. The decrease in *miR-124* observed in HD may diminish the post-translational repression of non-neuronal PTB form and promote its accumulation neurons [93].

Accumulation of glutamate after ischemic stroke is closely associated with the down-regulation of expression of glutamate transporter-1 (GLT-1) – indirect *miR-124* target in glial cells. In this case, neuronal *miR-124* increases astroglial GLT1 expression via exosome transfer. Thus, *miR-124* can be considered as a potential novel target for neuronal injuries upon cerebral ischemia [94]. Cell type-dependent targets, and in consequence phenotype driven by *miR-124*, provide clear evidence that supports the use of *miR-124* replacement or knock-down as a new therapeutic approach to boost endogenous brain repair mechanisms in a setting of neurodegeneration while inhibiting cancer progression.

### 5.5. miR-128 and Neural Stem Cells Fate

*MiR-128* is another microRNA abundant in healthy neurons and deregulated in both neurodegeneration and cancer [95]. Under-expression of *miR-128* in brain tumor and particularly in more aggressive ones such as glioblastoma and medulloblastoma was shown in many studies [96,97,98,99,100].

The expression of *miR-128* differs depending on cancer type but is consistently lost in GBM [101]. Both tumorigenicity and therapy resistance are diminished upon re-introduction of *miR-128* into glioblastoma cells [100,101,102]. *MiR-128* is one of the few microRNAs that shows a significant correlation with glioblastoma classification, with the most significant downregulation in mesenchymal tumors [103,104,105]. Perhaps its career in brain tumor started 10 years ago when we showed that *miR-128* targeted components of Polycomb repressive complexes 1 and 2 (PRC 1/2) – BMI1 and SUZ12, respectively. Such dual targeting thereby prevented partially redundant functions of these targets [102,103,106]. As epigenetic repression of transcription driven by PRCs is linked also to tumors beyond the brain, these microRNA-mRNA targets may have broad applicability in cancer research [107,108]. Our resent study has shown that *miR-128* targets also glioblastoma subtype-specific mRNAs that are relevant to the patient outcome. Such cell-specific targeting by inter-subtype transferring of microRNA by extracellular vesicle within single tumor provides a mechanistic explanation to how the gain/loss of *miR-128* contributes to dynamic bidirectional transitions between the subclasses within the single tumor [103]. Glioblastoma stem cells (GSC) share characteristics with neural progenitors cells (NPCs) [109], including the expression of neural stem cell markers, the capacity for self-renewal, long-term proliferation and the formation of neurospheres. However, GSCs differ from NPC in aberrant expression of differentiation markers, chromosomal abnormalities and tumorigenicity [110,111,112,113]. *MiR-128* control critical steps in committing toward neuronal lineage and maturing into terminally differentiated neurons. The importance of *miR-128* in the development of the mammalian brain and their loss in tumorigenesis has been shown [102,105,114,115,116,117].

Evaluation of microRNA expression in fetal, adult and AD hippocampi showed that alteration of *miR-128* contributes to neuronal dysfunction [118]. Reactive oxygen species (ROS) result in hyper-upregulation of *miR-128* in cultured neurons, which suggests the possibility that microRNA may mediate ROS’s pathogenic effects in AD [119]. *miR-128* is highly expressed in the hippocampus of AD patients relative to age-matched controls [120,121] and its expression is upregulated in the hippocampus in an intermediate stage of AD patients [120]. However, other studies have shown that a decrease in the *miR-128* level significantly reduced apoptosis and caspase-3 activity in AD model on primary mouse cortical neurons and Neuro2a cells. In this model, Aβ mediated toxicity was decreased by targeting PPAR-γ via inactivation of NF-κB [120]. These results were confirmed in a mouse model of AD, where a *miR-128* knockout weakened AD-like performances and reduced Aβ production and inflammatory responses by targeting PPARγ [122].

Multiple sclerosis (MS) is the most common immune-mediated disorder affecting the central nervous system. *MiR-128* has been shown to be overexpressed in brain immune cells, serving as a pro-inflammatory microRNA. Mechanistically, these effects were mediated by direct suppression of BMI1 and interleukin-4 expression, resulting in decreased GATA3 levels, and a T-cell cytokine shift [123].

Deficiency in *miR-128* leads to an increased excitability of dopamine D1 receptor-expressing neurons (D1 neurons) in the striatum and juvenile hyperactivity. It was shown that D1 neurons displaying decreased *miR-128-2* show increased expression levels of ERK network regulators and increased ERK2 activation. Increased *miR-128* expression reduces abnormal motor activities observed in chemically induced PD and epileptic seizures. These findings indicate *miR-128-*2 participation in these motor disorders [124]. Another study showed the protection of dopamine neurons from apoptosis driven by *miR-128* to be associated with upregulation of the expression of excitatory amino acid transporter 4 (EAAT4) in PD. The mechanism engaged by targeting the *miR-128* -AXIN1 inhibitory partner of EAAT4 [125]. *MiR-128* was also shown to be decreased in the frontal cortex of transgenic HD non-human primates and correspondingly in striatum samples analyzed from both pre-symptomatic and symptomatic human patients [126]. The protective role of *miR-128* against apoptosis induced by ischemia [127] together with its anti-proliferative function in cancer stem cells and pathological outcome of its hyper-expression in AD, underline not only cell type-specific function but also the importance of balanced expression. The inverse correlation of *miR-128* expression levels (loss in brain malignancies, upregulation in neurodegenerative disorders) underlines the importance of balanced expression within physiological range and suggests the opposite therapeutic approaches in both cases.

### 5.6. miR-210 Hypoxic Signaling and Niche

Hypoxia is a perilous feature of the glioblastoma microenvironment and has been associated with poor prognosis and resistance to various therapies. Hypoxia also is associated with the downregulated profile of gene expression, so it was speculated that this is associated with widespread upregulation of microRNA. However, with few exceptions, it is not a case [128]. These include highly conserved, hypoxia-regulated microRNA - *miR-210*. Its expression is modulated via a hypoxia response element (HRE) within the promoter [129,130]. *MiR-210* has been upregulated under hypoxic conditions in all tissues and cell types examined, and it is thus considered to be the master microRNA regulator of the hypoxia response. It is involved in numerous functions, including mitochondrial respiration, DNA repair, cell proliferation, and angiogenesis [131,132,133]. *MiR-210* increases transcriptional activity of the hypoxia-inducible factor (HIF1A) and its target genes—vascular endothelial growth factor (VEGF) and carbonic anhydrase 9 (CAIX). Hypoxic survival promoted by *miR-210-3p* was also associated with chemo-resistance in GBM cells by targeting a negative regulator of hypoxic response, HIF3A [132]. Recently NeuroD2, the levels of which are tightly regulated by *miR-210*, was shown to act as a tumor suppressor and prognostic biomarker in glioblastoma [134]. As *miR-210* expression is associated with hypoxia, it’s targeting of NeuroD2 links undifferentiation status of cells in a hypoxic niche with the pathogenesis of glioblastoma. However, *miR-210* is detected not only in tumor cells but also in the tumor microenvironment including immune cells, highlighting the necessity of using complementary approaches to account for the cell-specific context of microRNA expression [135].

Ectopic overexpression of *miR-210* has been shown to stimulate angiogenesis and proliferation of both embryonic and adult neural progenitors within the mouse brain SVZ [136,137]. It was also implicated in various models of epilepsy, as well as in vitro, in an *N*-methyl-D-aspartate (NMDA) receptor-dependent manner following the exposure of primary rat neurons to soluble amyloid β, a pathogenic component of AD. These results suggest a strong correlation of *miR-210* regulation with neuronal activation in rodent models, as well as a likely conserved role in learning and memory in mammalian systems. Upregulation of *miR-210* in a PD model reduces BDNF production and contributes to the DA neurons degeneration [138].

*MiR-210* is related to vascular remodeling after ischemic injuries and its presence increases tissue perfusion and capillary density after renal ischemia/reperfusion (I/R) injury and myocardial injury [139,140,141]. Significantly higher *miR-210* levels were observed in patients with good outcomes after a stroke than in those with poor outcomes. This association can be linked to the effect of *miR-210* on endothelial cells. It was shown that the overexpression of *miR-210* promotes the migration of endothelial cells and the formation of vascular-like structures, whereas inhibiting *miR-210* expression results in a decrease of endothelial cell growth and migration, inhibition of the formation of vascular-like structures and induces apoptosis [142]. Recently, it was reported that *miR-210* promotes neovascularization and neural precursor cells migration toward the infarct area after cerebral ischemia via the SOCS1-STAT3-VEGF-C pathway, facilitating nerve repair [143].

Recent data has shown that suppression of *miR-210* significantly reduces the expression of pro-inflammatory cytokines (TNF-α, IL-1β, IL-6) and chemokines (CCL2 and CCL3) but did not affect anti-inflammatory factors like TGF-β or IL-10 [144].

It was shown that HI significantly increases the *miR-210* level in the rat pups’ brain and that glucocorticoid receptor (GR) is a novel target of *miR-210* [145,146]. *MiR-210* effect of down-regulation of GR exacerbated HI evoked brain injury in rat pups. Ma and colleagues [147] also reported that increased *miR-210* is involved in the disruption of the blood-brain barrier. On the other hand, Zhao [147] recently reported the down-regulated expression of *miR-210* after HI connecting this with the development of HI brain edema in neonatal rats. Moreover, pre-treatment with *miR-210* mimic significantly improved HI-induced edema reducing water content in brain tissue.

It was shown that intracerebroventricular or intranasal application of *miR-210*-LNA to silencing *miR-210* resulted in a neuroprotective effect on neonatal brain HI insult [145]. The current finding that pre-treatment with *miR-210* inhibitor significantly reduced HI-induced brain infarct size indicates on a functional significance of *miR-210* in the pathophysiology of HI-induced brain injury in the developing brain. Moreover, *miR-210* was reported to be involved in the regulation of angiogenesis in response to ischemic brain injury [148] and it was also shown that up-regulation of *miR-210* protects cells from hypoxia-induced apoptosis [128]. It thus seems that the tissue type and the differences in insult character determine whether *miR-210* plays a protective or detrimental.

## 6. More about microRNA in Neuropathology

Even though numerous microRNAs were found to be functional in neurodegenerative disorders but not in cancer, we should probably keep track of them all for the benefit of a therapeutic strategy. Deregulation of a single microRNA can drive subsequent deregulation not only of its direct targets but also indirect coding and non-coding RNAs, including other microRNAs. In addition to microRNAs reviewed in the context of neurodegeneration (*miR-9, miR-21, miR-22, miR-25, miR-26, miR-29, miR-30, miR-34, miR-98, miR-106, miR-107, miR-124, miR-125, miR-127, miR-128, miR-132, miR-134, miR-141, miR-153, miR-181, miR-183, miR-186, miR-193, miR-196, miR-200, miR-210, miR-212,miR-221, miR-223, miR-320, miR-365, miR-378, miR-494, miR-505, miR-512, miR-592, miR-133b, miR-146a, miR-let7*) [15,16,149,150,151] or brain cancer (*miR-21, miR-17–92, Let-7, miR-10b, miR-34a, miR-7, miR-124-3p, miR-124-5p, miR-137, miR-326, miR-99a, miR-524-5p, miR-328, miR-128, miR-101, miR-302–367, miR-143, miR-145, miR-218, miR-93, miR-125b, miR-451, miR-222, miR-339, miR-148a, miR-181d, miR-297*) [13,14,29,152], recently others have been identifies as potential therapeutic targets.

In case of neurodegeneration, oxidative stress dependent neurotoxicity and apoptosis was linked with following microRNAs: *miR-98*, *miR-132*, *miR-153*, *miR-200a*, *miR-186*, *miR-221*, *miR-196a*, *miR-22*, *miR-107*, *miR-223*, *miR-320*, *miR-125b*, *miR-181*, *miR-365*, *miR-193*, *miR-49*, *miR-378*, *miR-25*, *miR-30d-5p*, *miR-26b*, *miR-592-5p*, *miR-153*, *miR-155*, *miR-212*, *miR-183/96/182* cluster, *miR-146a* [94,151,153,154,155,156,157,158,159,160,161,162,163,164,165,166,167,168,169,170,171,172,173,174,175,176,177,178,179,180,181,182,183,184,185,186]. MicroRNAs that are linked to the therapy resistance and sensitivity are the ones engaged in cancer cell survival. In glioblastoma, these microRNAs are currently under investigation: *miR-183/96/182*, *miR-143*, *miR-449a*, *miR-107*, *miR-122*, *miR-152* and *miR-217 miR-200a*, *miR-873*, *let-7b*, *miR-136*, *miR-139*, *miR-1*, *miR-451*, *miR-143*, *miR-603*, *miR-100*, *miR-96*, *miR-93-5p*, *miR-146b*, *miR-183*, *miR-378*, *miR-125b/20b*, *miR-338-5b*, *miR-203* (Table 2) [187,188,189,190,191,192,193,194,195,196,197,198,199,200,201,202,203,204].

## 7. Concluding Remarks

**Figure 1:**
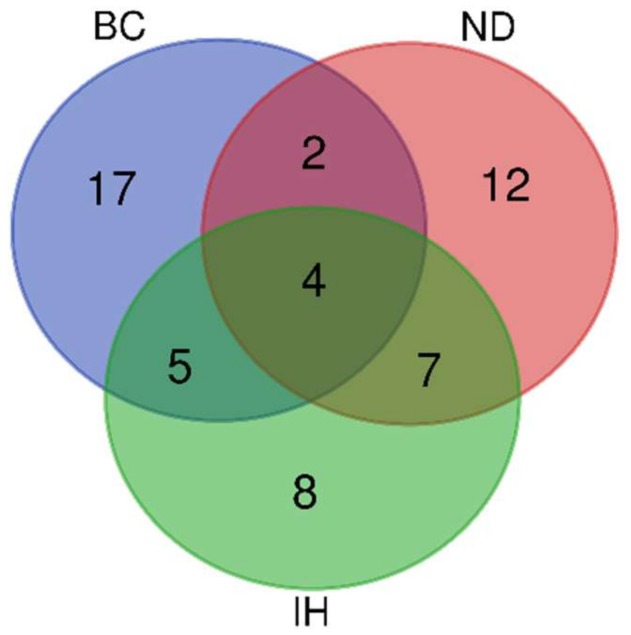
Gene Venn showing microRNA under investigation in selected pathologies of the brain (based on Table 2) (Brain Cancer (BC), Neurodegenerative Disorders (ND) Ischemia (IH)). *MiR-21*, *let-7*, *miR-210* and *miR-128* are common microRNAs.

We have discussed the importance of microRNA in brain pathologies. Changes of microRNAs profiles can serve as biomarkers of the functional status of a healthy brain, as well as of progression of CNS diseases. Interestingly, it seems that the majority of microRNAs have inverse effects in tumorigenic events and neurodegenerative processes. For example, *miR-124* alleviates cell death in AD, while increases cell death in glioblastoma. This might be due to different targets involved, depending on the context of diseases and cell types. Similarly, *miR-128* that is lost in glioblastoma is elevated in such neurodegenerative processes as MS or AD. Another example is *miR-21* that is elevated in glioblastoma where it promotes cell growth [205], while its ectopic upregulation in undifferentiated neuroblastic cells enhances neuronal differentiation [206]. Regions of the brain with robust neurogenesis (such as SVZ and hippocampus) showed altered expression of neural fate microRNAs in both neurodegeneration and cancer [207,208]. As neural differentiation of stem and progenitor cells is associated with apoptotic cell death [209], the therapeutic use of differentiation [208] is a promising approach as we have recently shown [207]. Moreover, the regulation of numerous targets by microRNAs (summarized in Table 1) makes them good candidates for therapeutic intervention. The research on microRNA targeting is in the midst of a volte-face. Thousands of research projects have pushed ahead with the discovery of microRNA targeting for medical purposes. However, for decades we have argued that we need just a good candidate with a relevant target. Now we think that this is less about small RNA reintroduction that has been overstated but more about the rigorous studies of microRNA in relevant models. Currently, only a few microRNAs, such as *miR-128*, are poised to replace single gene therapies for the treatment of these brain diseases shortly (Figure 1).

## Figures and Tables

**Table 1:** Functional microRNAs deregulated in brain disorders.

microRNA	Disorders	Target
*miR-9*	GBM	FOXP1, CREB, NF1, STMN1 [41,42,43,44,45]
	PD, AD	SIRT1, BACE1, BDNF, Bcl-2 [49]
	HD	REST, CoREST [46]
*miR-29*	GBM	SCAP/SREBP-1 [37], PTEN [55]
	AD, HD	VDAC, BACE [57,58]
	PD	PTEN [56]
*miR-34abc*	GBM	PDGFRA [64]
	PD	BCL [74], DJ1, Parkin [73]
	AD	Cyclin D1, SORT1 [75,77], p53 [76]
*miR-124*	GBM	SCP1, PTPN12, SNAIL2, ROCK1 [85,86,87], IQGAP1, LAMC1, ITGB1 [84], TWIST [87], TEAD1, MAPK14/p38α [88]
	PD	SOX9, JAGGED1 [89], BIM [90],
	HD	PTB, REST [91,92]
	Ischemia	GLT1/EAAT2 [93]
*miR-128*	GBM	BMI1, ABCC5, E2F5 [98,101], RTKs, EGFR, PDGFRα [102,104], SUZ12 [106]
	AD	ROS [118], PPARγ/NF-ĸB [119]
	PD	ERK2 [123], AXIN1/EAAT4 [124]
	MS	BIM1, IL-4, GATA3 [122]
	Ischemia	p38 MAPK [126]
*miR-210*	GBM	HIF1α, HIF3A, VEGF, CAIC [131], NeuroD2 [134]
	PD	BDNF [145]
	Ischemia	VEGF [138,139,140], SOCS1-STAT3-VEGF [141], TNFα, IL-1B, IL-6, CCL 2/3 [142], GR [143], occluding, β-catenin [144]. Notch 1 [145]

**Table 2:** List of microRNAs deregulated in brain disorders.

Disorders	microRNAs
Brain Cancer (BC)	*miR-9, miR-21, miR-17–92, Let-7, miR-10b, miR-34a, miR-7, miR-124-3p, miR-124-5p, miR-137, miR-326, miR-99a, miR-524-5p, miR-328, miR-128, miR-101, miR-302–367, miR-143, miR-145, miR-218, miR-93, miR-125b, miR-451, miR-222, miR-339, miR-148a, miR-181d, miR-210, miR-297*
Neurodegenerative Disorders (ND)	*miR-7, miR-9, miR-17-p, miR-21, miR-22, miR-26, miR-29, miR-30a-5p, miR-34, miR-101, miR-107, miR-124, miR-128, miR-133, miR-146, miR-153, miR-132, miR-196a, miR-197, miR-210, miR-200a, Let-7, miR-221, miR-494, miR-512*
Ischemia (IH)	*Let-7, miR-19, miR-21, miR-26, miR-30d-5p, miR-34, miR-107, miR-96, miR-124, miR-125b, miR-128, miR-132, miR-136, miR-137, miR-181d, miR-182, miR-200a, miR-210, miR-218, miR-223, miR-320, miR-328, miR-494, miR-592-5p,*
